# Antidepressant Effects of TrkB Ligands on Depression-Like Behavior and Dendritic Changes in Mice After Inflammation

**DOI:** 10.1093/ijnp/pyu077

**Published:** 2015-01-26

**Authors:** Ji-chun Zhang, Jin Wu, Yuko Fujita, Wei Yao, Qian Ren, Chun Yang, Su-xia Li, Yukihiko Shirayama, Kenji Hashimoto

**Affiliations:** Division of Clinical Neuroscience, Chiba University Center for Forensic Mental Health, Chiba, Japan (Drs Zhang, Wu, Yao, Ren, Yang, Li, Shirayama, Hashimoto, and Ms Fujita); National Institute of Drug Dependence, Peking University, Beijing, China (Dr Li); Department of Psychiatry, Teikyo University Chiba Medical Center, Chiba, Japan (Dr Shirayama).

**Keywords:** BDNF-TrkB signaling, hippocampus, inflammation, nucleus accumbens, prefrontal cortex

## Abstract

**Background:**

Brain-derived neurotrophic factor (BDNF) and its receptor, tropomyosin-related kinase B (TrkB), signaling represent potential therapeutic targets for major depressive disorder. The purpose of this study is to examine whether TrkB ligands show antidepressant effects in an inflammation-induced model of depression.

**Methods:**

In this study, we examined the effects of TrkB agonist 7,8-dihydroxyflavone (7,8-DHF) and TrkB antagonist ANA-12 on depression-like behavior and morphological changes in mice previously exposed to lipopolysaccharide (LPS). Protein levels of BDNF, phospho-TrkB (p-TrkB), and TrkB in the brain regions were also examined.

**Results:**

LPS caused a reduction of BDNF in the CA3 and dentate gyrus (DG) of the hippocampus and prefrontal cortex (PFC), whereas LPS increased BDNF in the nucleus accumbens (NAc). Dexamethason suppression tests showed hyperactivity of the hypothalamic-pituitary-adrenal axis in LPS-treated mice. Intraperitoneal (i.p.) administration of 7,8-DHF showed antidepressant effects on LPS-induced depression-like behavior, and i.p. pretreatment with ANA-12 blocked its antidepressant effects. Surprisingly, ANA-12 alone showed antidepressant-like effects on LPS-induced depression-like behavior. Furthermore, bilateral infusion of ANA-12 into the NAc showed antidepressant effects. Moreover, LPS caused a reduction of spine density in the CA3, DG, and PFC, whereas LPS increased spine density in the NAc. Interestingly, 7,8-DHF significantly attenuated LPS-induced reduction of p-TrkB and spine densities in the CA3, DG, and PFC, whereas ANA-12 significantly attenuated LPS-induced increases of p-TrkB and spine density in the NAc.

**Conclusions:**

The results suggest that LPS-induced inflammation may cause depression-like behavior by altering BDNF and spine density in the CA3, DG, PFC, and NAc, which may be involved in the antidepressant effects of 7,8-DHF and ANA-12, respectively.

## Introduction

Accumulating evidence suggests that inflammatory processes play a role in the pathophysiology of major depressive disorder (MDD; Dantzer et al., 2008; Hashimoto, 2009; Miller et al., 2009; Raison et al., 2010). Peripheral administration of the bacterial endotoxin lipopolysaccharide (LPS) induces depression-like behavior in rodents after the induction of inflammation (Dantzer et al., 2008; O’Connor et al., 2009). Depression-like behavior and alterations in serum pro-inflammatory cytokines induced by LPS can be blocked by anti-inflammatory drugs (de Paiva et al., 2010) and antidepressants, including selective serotonin reuptake inhibitors (SSRIs) and serotonin and norepinephrine reuptake inhibitors (SNRIs; Ohgi et al., 2013). Meta-analysis shows higher blood levels of pro-inflammatory cytokines in drug-free MDD patients, compared with healthy controls (Dowlati et al., 2010). A study on post-mortem brains revealed elevated gene expression of pro-inflammatory cytokines in the frontal cortices of participants with a history of MDD (Shelton et al., 2011). These findings suggest that both peripheral and central inflammations are associated with depressive symptoms, and it is therefore highly plausible that anti-inflammatory drugs could ameliorate depressive symptoms in these patients.

Multiple lines of evidence link brain-derived neurotrophic factor (BDNF), its specific receptor tropomyosin-receptor-kinase B (TrkB), and subsequent mammalian target of rapamycin complex 1 (mTORC1) signaling to the pathophysiology of MDD, as well as the therapeutic mechanisms of antidepressants (Nestler et al., 2002; Hashimoto et al., 2004; Duman and Monteggia 2006; Martinowich et al., 2007; Hashimoto, 2010; Hashimoto, 2011, 2013a; Duman et al., 2012; Castrén, 2014; Lindholm and Castrén, 2014). In learned helplessness models of depression, a single infusion of BDNF into the dentate gyrus (DG) and CA3 pyramidal cell layers of the hippocampus show long-lasting antidepressant effects (Shirayama et al., 2002). A viral-mediated gene transfer approach found that BDNF in the DG might be essential for mediating the therapeutic effect of antidepressants (Adachi et al., 2008). A study showed that heterozygous BDNF^+/Met^ mice, carrying the human BDNF Val66Met polymorphism, exhibited decreased BDNF levels and apical dendritic spine density in the prefrontal cortex (PFC) after stress, which caused depression-like behavior (Yu et al., 2012). Furthermore, loss of BDNF in the forebrain attenuated the actions of an antidepressant (Monteggia et al., 2004), and responses elicited by antidepressants were lost in mice with either reduced brain BDNF levels or inhibited TrkB signaling (Saarelainen et al., 2003; Monteggia et al., 2007). In contrast, Nestler’s group demonstrated that BDNF in the ventral tegmental area (VTA)-nucleus accumbens (NAc) pathway is required for depression onset (Eisch et al., 2003; Berton et al., 2006; Nestler and Carlezon, 2006; Krishnan et al., 2007). This suggests that BDNF acts within the VTA-NAc pathway, inducing a depression-like phenotype (Eisch et al., 2003; Berton et al., 2006; Nestler and Carlezon, 2006; Krishnan et al., 2007), whereas it produces antidepressant-like effects in the hippocampus and PFC (Nestler et al., 2002; Shirayama et al., 2002; Duman and Monteggia, 2006). Currently, there are no reports on the relationship between BDNF-TrkB signaling and dendritic changes in the hippocampus, PFC, and NAc, and the antidepressant action of TrkB ligands.

This study aims to examine whether TrkB ligands exert antidepressant effects on inflammation-induced depression-like behavior in mice. Here, we used 7,8-dihydroxyflavone (7,8-DHF), a novel TrkB agonist (Jang et al., 2010; Andero et al., 2011), and ANA-12, a novel TrkB antagonist (Cazorla et al., 2011), as pharmacological tools. We examined the effects of these compounds on depression-like behavior, BDNF protein levels, TrkB phosphorylation, and spine density in selected brain regions, including the hippocampus, PFC, and NAc.

## Methods and Materials

### Animals

Male, adult C57BL/6 mice, aged 8 weeks (body weight 20–25g, Japan SLC, Inc.) were used in experiments. Animals were housed under controlled temperatures and 12 hour light/dark cycles (lights on between 07:00–19:00h), with *ad libitum* food and water. A total of 306 mice were used in the experiment. All experiments were carried out in accordance with the Guide for Animal Experimentation of Chiba University. The procedures of this animal experiment were approved by the Chiba University Institutional Animal Care and Use Committee.

### Drug Administration

On the day of injection, fresh solutions were prepared by dissolving compounds in sterile endotoxin-free isotonic saline. Lipopolysaccharide (LPS, 0.5mg/kg; L-4130, serotype 0111:B4, Sigma-Aldrich) was administered intraperitoneally (i.p.). 7,8-Dihydroxyflavone (7,8-DHF; Catalog number: D1916) and 5,7-dihydroxyflavone (5,7-DHF: Catalog number: C1652) were purchased from Tokyo Chemical Industry (Supplementary Figure 1). 7,8-DHF (1, 3, or 10mg/kg, i.p.) and 5,7-DHF (10mg/kg, i.p.) were prepared in a vehicle of 17% dimethylsulfoxide in phosphate-buffered saline (Ren et al., 2013 2014). ANA-12, N2-(2-{[(2-oxoazepan-3-yl) amino]carbonyl}phenyl)benzo[b]thiophene-2-carboxamide (0.5mg/kg, i.p., Catalog number: BTB06525SC, Maybridge; Supplementary Figure 1), was dissolved in 1% dimethylsulfoxide in physiological saline. Paroxetine (as the hydrochloride salt, at 10mg/kg, i.p.) and venlafaxine (as the hydrochloride salt, at 10mg/kg, i.p.; Wako Pure Chemical Ltd.) were dissolved in physiological saline. Rapamycin (0.2 nmol/L in 2 µL, Calbiochem-Novabiochem) was administered intracerebroventricularly (i.c.v.), after the mice were anesthetized with pentobarbital (5mg/kg). The dose of rapamycin was selected as previously reported (Li et al., 2010 2011). The doses of 7,8-DHF and ANA-12 were also selected as previously reported (Ren et al., 2013 2014; Cazorla et al., 2011).

## Behavioral Tests

On day 1, saline (10 ml/kg) or LPS (0.5 mg/kg) was injected i.p. On day 2, all behavioral tests were performed in the following order: the locomotion test (24–25 hours after LPS injection), tail suspension test (TST; 27 hours after LPS injection), and forced swimming test (FST; 29 hours after LPS injection). All behavioral tests were performed as following: Locomotion: the mice were placed in experimental cages (length × width × height: 560 × 560 × 330 mm). Locomotor activity of mice was counted by the SCANETMV-40 (MELQUEST Co., Ltd., Toyama, Japan), and cumulative exercise was recorded for 60 minutes. Cages were cleaned between testing session. Tail suspension test (TST): The mice were taken from their home cage and a small piece of adhesive tape was placed approximately 2 cm from the tip of their tail. A single hole was punched in the tape and mice were hung individually, on a hook. The immobility time of each mouse was recorded for 10 minutes. Mice were considered immobile only when they hung passively and completely motionless. Forced swimming test (FST): The mice were placed individually in a cylinder (diameter: 23 cm; height: 31 cm) containing 15 cm of water, maintained at 23 ± 1°C. Animals were tested in an automated forced-swim apparatus using SCANETMV-40 (MELQUEST Co., Ltd., Toyama, Japan). Immobility time was calculated from activity time as (total) – (active) time, using the apparatus analysis software. Cumulative immobility time was scored for 6 minutes during the test. Mice were put into the test room 30 minutes before behavioral tests commenced. All tests were performed between 9:00 am–17:00 pm in a quiet room.

### Surgery and Bilateral Injection of ANA-12 into NAc

Mice were anesthetized with pentobarbital (5mg/kg), and placed in a stereotaxic frame. Microinjection needles were placed bilaterally into the NAc shell (+1.7 AP, ±0.75 ML, -3.6 DV) (Paxinos and Watson, 1998). Twenty-four hours after surgery, LPS (0.5mg/kg) or saline (10ml/kg) was injected i.p. Twenty-three hours after injection of LPS (or saline), ANA-12 (0.1 nmol/L, 0.1 μL/min for 5min) or vehicle was injected bilaterally. Behavioral evaluation was performed 4 and 6 hours after the final infusion (Figure 3B).

### Dexamethasone Suppression Test

Between 09:00 and 10:00 hours, dexamethasone (DEX: Wako Pure Chemical Co., 0.1mg/kg, i.p.) was injected into mice, 23 hours after i.p. administration of saline or LPS (0.5mg/kg). Mice were anesthetized with pentobarbital (50mg/kg), and blood was collected 6 hours after DEX injections. Serum levels of corticosterone were measured using an Assay MAX corticosterone ELISA kit (St. Charles, MO).

### Western Blot Analysis

Mice were killed by cervical dislocation and brains were rapidly removed from the skull. Approximately 1-mm-thick coronal sections were cut and bilateral tissue punches of the CA1, CA3 and dentate gyrus (DG) of the hippocampus, prefrontal cortex (PFC) and NAc (Paxinos and Watson, 1998) were dissected on ice using a SZ-LED Kenis light microscope (Osaka, Japan), and stored at -80°C. The rostral faces of the coronal sections were cut approximately 1.98 mm from the bregma for the PFC, and the tissue was collected. Tissue samples were homogenized in Laemmli lysis buffer. Aliquots (10 μg) of protein were measured using the DC protein assay kit (Bio-Rad, Hercules, CA, USA), and incubated for 5 min at 95 °C, with an equal volume of 125 mM Tris/HCl, pH 6.8, 20% glycerol, 0.1% bromophenol blue, 10% β-mercaptoethanol, 4% sodium dodecyl sulfate, and subjected to sodium dodecyl sulfate polyacrylamide gel electrophoresis, using 10% mini-gels (Mini-PROTEAN® TGX™ Precast Gel; Bio-Rad). Proteins were transferred onto polyvinylidenedifluoride (PVDF) membranes using a Trans Blot Mini Cell (Bio-Rad). For immunodetection, the blots were blocked with 2% BSA plus 5% nonfat dry milk in TBST (TBS + 0.1% Tween-20) for 1 h at room temperature (RT), and kept with primary antibodies overnight at 4°C. The following primary antibody was used: BDNF (1: 200, Santa Cruz Biotechnology, Inc., CA), Phosphor-TrkB (Tyr 706) (1:200, Santa Cruz Biotechnology, Inc., CA) and TrkB (80E3) (1:1000, Cell Signaling Technology, MA, USA). The next day, blot were washed three times in TBST and incubated with horseradish peroxidase conjugated anti-rabbit antibody (1:10000) or goat anti-rabbit antibody (1:2000) or anti-rabbit antibody (1:1000) for 1hour, at RT. After final three washes with TBST, bands were detected using enhanced chemiluminescence (ECL) plus the Western Blotting Detection system (GE Healthcare Bioscience). The blots then were incubated in the stripping buffer (2% SDS, 100 mM β-mercaptoethanol, 62.5 mM Tris/HCL PH 6.8) for 30 min at 60°C followed by three time washed with TBST. The stripped blots were kept blocking solution for 1 hour and incubated with the primary antibody directed against TrkB protein or β-Actin. Images were captured with a Fuji LAS3000-mini imaging system (Fujifilm, Tokyo, Japan), and immunoreactive bands were quantified.

### Golgi Staining

Golgi staining was performed using the FD Rapid GolgiStainTM Kit (FD Neuro Technologies, Inc., Columbia, MD, USA), following the manufacturer’s instructions. Twenty seven hours after i.p. administration of LPS (0.5 mg/kg) or saline (10 ml/kg), animals were deeply anesthetized with sodium pentobarbital, and brains were removed from the skull and rinsed in double distilled water. Brains were immersed in the impregnation solution, made by mixing equal volumes of Solution A and B, overnight and then stored in fresh solution, for 2 weeks in the dark. Brains were transferred into Solution C overnight and then stored in fresh solution at 4°C for 1 week, in the dark. Coronal brain sections (100 µm thickness) were cut on a cryostat (3050S, Leica Microsystems AG, Wetzlar, Germany), with the chamber temperature set at -20°C. Each section was mounted in Solution C, on saline-coated microscope slides. After absorption of excess solution, sections were dried naturally, at room temperature. Dried sections were processed following the manufacturer’s instructions. Briefly, images of dendrites within CA1, CA3, and DG of the hippocampus, PFC and NAc were captured using a 100× objective with a Keyence BZ-9000 GenerationⅡmicroscope (Osaka, Japan). Spines were counted along CA1, CA3, DG, PFC and NAc dendrites starting from their point of origin from the primary dendrite, as previously reported (Milatovic et al., 2010). For spine density measurements, all clearly evaluable areas containing 50-100 µm of secondary dendrites from each imaged neuron were used. To determine relative spine density, spines on multiple dendritic branches from a single neuron were counted to obtain an average spine number per 10 µm. For spine number measurements, only spines that emerged perpendicular to the dendritic shaft were counted. Three neurons per section, three sections per animal and six animals were analyzed. The average value for each region, in each individual was obtained. These individual averages were then combined to yield a grand average for each region.

### Statistical Analysis

The data are shown as the mean ± standard error of the mean (SEM). Analysis was performed using PASW Statistics 20 (formerly SPSS Statistics; SPSS). All data, including results for locomotion, TST, FST, BDNF protein, p-TrkB, total TrkB, Golgi staining, and corticosterone, were analyzed using one-way analysis of variance (ANOVA), followed by post hoc LSD test or student’s *t*-test. The *p*-values of less than 0.05 were considered statistically significant.

## Results

### Effects of LPS on BDNF Levels in the Brain and DEX Suppression Test

**Figure 1.**
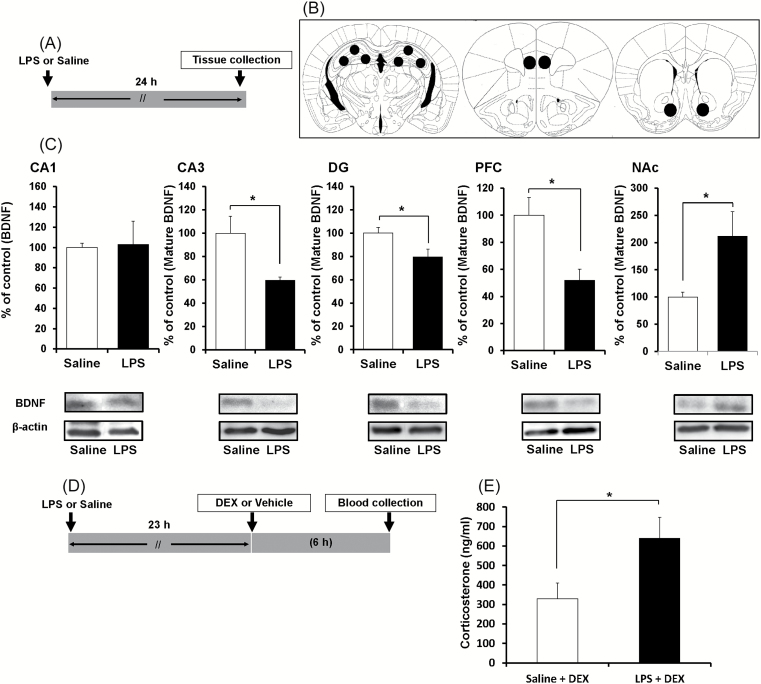
BDNF protein in the brains of control and LPS-treated mice. (A): The schedule of treatment. (B): Brain regions (CA1, CA3, DG, PFC, NAc) for Western blot analysis of BDNF. (C): Western blot analysis of BDNF in the CA1, CA3, and DG of the hippocampus, the PFC, and the NAc. The value was expressed as a percentage of that of saline-treated mice. Values represent the mean ± standard error of the mean (SEM; n = 6). *p < 0.05 compared with saline treated groups (student’s *t*-test). (D and E): Dexamethasome (DEX) suppression test. (D): The schedule of treatment. (E): Blood sample was collected 6 hours after injection of DEX, and serum corticosterone was measured by ELISA. Data represent mean ± SEM (n = 11–12 mice per group). *p < 0.05 compared with saline-treated groups (student’s t-test). LPS: lipopolysaccharide; DG: dentate gyrus; PFC: prefrontal cortex; NAc: nucleus accumbens.

First, we examined whether the levels of BDNF are altered in mouse brains after LPS administration. A single dose of LPS significantly decreased BDNF protein in the CA3 (*p* = 0.020), DG (*p* = 0.033), and PFC (*p* = 0.010), but not in the CA1 (*p* = 0.900; Figure 1C). By contrast, a single dose of LPS significantly (*p* = 0.036) increased BDNF protein in the NAc (Figure 1C).

The DEX suppression test has been used effectively to quantify dysregulation of the hypothalamic-pituitary-adrenal (HPA) axis (Kathol et al., 1989; Pariante et al., 2001). We found that serum levels of corticosterone in LPS-treated mice were significantly (*p* = 0.037) higher than in saline-treated mice six hours after DEX injections (0.1mg/kg; Figure 1D and E), suggesting that the HPA axis was hyperactive in the LPS-treated mice.

### Antidepressant Effects of 7,8-DHF on LPS-Induced Depression-Like Behavior in Mice

**Figure 2.**
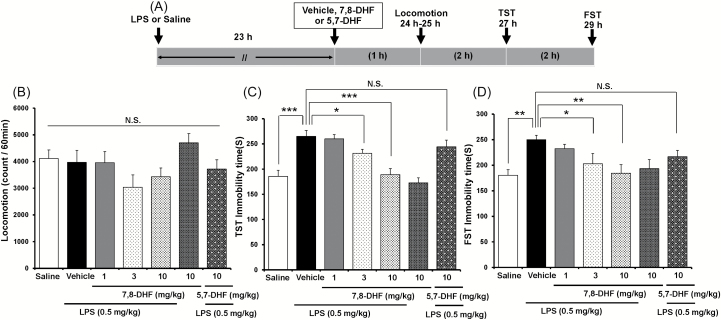
Effects of 7,8-DHF and 5,7-DHF on LPS-induced depression-like behavior. (A) The schedule of treatment and behavioral evaluations. (B) Locomotion, (C) TST, and (D) FST tests. Values represent the mean ± standard error of the mean (n = 8–11). *p < 0.05, **p < 0.01, ***p < 0.001 compared with the vehicle + LPS group. N.S.: not significant. 7,8-DHF: 7,8-dihydroxyflavone; 5,7-DHF: 5,7-dihydroxyflavone; LPS: lipopolysaccharide; TST: tail suspension test; FST: forced swimming test.

The FST and TST are the most widely used behavioral assays for detecting potential antidepressant-like activity in mice. To examine the therapeutic effects of 7,8-DHF on LPS-induced depression-like behaviors, 7,8-DHF was administered 23 hours after administration of LPS (0.5mg/kg, i.p.; Figure 2A). No effect was observed in spontaneous locomotion in vehicle and LPS-treated mice [one-way ANOVA, locomotion: F(6, 68) = 1.896, *p* = 0.96; Figure 2A]. In the TST and FST, 7,8-DHF (1.0, 3.0, or 10mg/kg, i.p.) significantly attenuated the increase in immobility time observed in mice after LPS administration, in a dose-dependent manner [one-way ANOVA; TST (c): F(6, 71) = 12.686, *p* < 0.001; FST (d): F(6, 64) = 3.210, *p* = 0.009; Figure 2C and D]. However, 7,8-DHF (10mg/kg, i.p.) alone did not affect immobility time during the TST and FST in control mice (Figure 2C and D).

5,7-DHF (Chrysin), a stereoisomer of 7,8-DHF (Supplementary Figure 1), is approved for bodybuilding use in humans, and possesses no TrkB agonist properties. Unlike the antidepressant effects seen with 7,8-DHF (10mg/kg, i.p.), 5,7-DHF (10mg/kg, i.p.) altered neither LPS-induced depression-like behavior in mice (TST and FST) nor spontaneous locomotion (Figure 2B–D; locomotion: *p* = 0.643; TST: *p* = 0.187; FST: *p* = 0.092). Furthermore, neither the SSRI paroxetine (10mg/kg, i.p.) nor the SNRI venlafaxine (10mg/kg, i.p.) altered LPS-induced depression-like behavior in mice (TST and FST; Supplementary Figure 2).

### Role of TrkB Signaling on the Antidepressant Effects of 7,8-DHF and ANA-12

**Figure 3.**
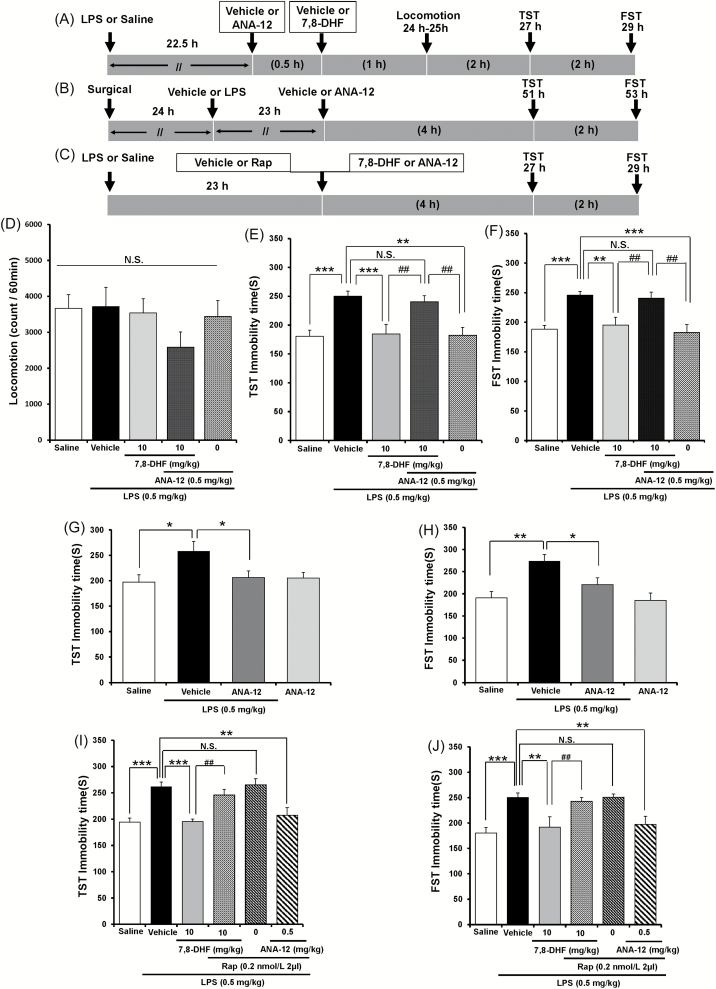
Role of TrkB and mTORC1 in the antidepressant action of 7,8-DHF and ANA-12 on LPS-induced depression-like behavior. (A–C) The schedule of treatment and behavioral evaluations. (D) Locomotion, (E) TST, and (F) FST tests. Values represent the mean ± standard error of the mean (SEM). n = 8–10 for D, E, and F. **p < 0.01, ***p < 0.001 compared with the LPS + vehicle group. ^##^ *p* < 0.01 compared with the LPS + 7,8-DHF + ANA-12 group. N.S.: not significant. (G) TST and (H) FST. Bilateral infusion of ANA-12 into the NAc significantly attenuated the increase of immobility time of LPS-treated mice. Values represent the mean ± SEM. n = 7 for (G) and (H). *p < 0.05, **p < 0.01 compared with the LPS + vehicle group. (I): TST, (J): FST. I.c.v. infusion of rapamycin significantly blocked the decrease of immobility time of LPS-treated mice by 7,8-DHF. In contrast, i.c.v. infusion of rapamycin did not affect the decrease of immobility time of LPS-treated mice by ANA-12. Values represent the mean ± SEM (n = 8 – 10). **p < 0.01, ***p < 0.001 compared with the LPS + vehicle group. ^##^ *p* < 0.01 compared with the LPS + 7,8-DHF group. N.S.: not significant. TrkB: tropomyosin-receptor-kinase B; mTORC1: mammalian target of rapamycin complex 1; 7,8-DHF:7,8-dihydroxyflavone; TST: tail suspension test; FST: forced swimming test; NAc: nucleus accumbens; i.c.v.: intracerebroventricular.

To assess the role of TrkB on the mechanistic action of 7,8-DHF, we examined the effects of ANA-12, a novel TrkB antagonist (Cazorla et al., 2011), on LPS-induced depression-like behavior (Figure 3A and B). One-way ANOVA showed statistical results for the locomotion [Figure 3D; F(4, 44) = 1.088, *p* = 0.375], TST [Figure 3E; F(4, 46) = 8.231, *p* < 0.001], and FST [Figure 3F; F(4, 46) = 7.703, *p* < 0.001]. ANA-12 did not alter locomotion in LPS-treated mice (Figure 3D). Furthermore, pretreatment with ANA-12 (0.5mg/kg, 30 minutes before injection of 7,8-DHF) significantly blocked the antidepressant effects of 7,8-DHF in mice treated with LPS (Figure 3E and F). Interestingly, ANA-12 (0.5mg/kg, i.p.) alone significantly attenuated increased immobility time in LPS-treated mice, suggesting antidepressant activity of ANA-12 (TST: p = 0.003; FST: *p* < 0.001; Figure 3E and F).

Next, we looked at the effects of direct ANA-12 infusion into the NAc on LPS-treated mice, since levels of BDNF in the NAc were increased after LPS administration (Figure 3B). One-way ANOVA showed that statistical significance for TST [Figure 3G; F (3, 27) = 3.346, p = 0.036] and FST [Figure 3H; F (3, 27) = 7.056, p = 0.001]. Bilateral injection of ANA-12 (0.1 nmol/L, 0.1 μL/min for 5min) into the NAc significantly attenuated increased immobility time in LPS-treated mice (TST: *p* = 0.024; FST: *p* = 0.022; Figure 3G and H). This implies an ANA-12 antidepressant effect, mediated through the blockade of TrkB signaling, in the NAc of these mice.

### Role of mTORC1 Signaling on the Mechanistic Action of 7,8-DHF

Recent studies highlight a role for mammalian target of rapamycin complex 1 (mTORC1) signaling in the rapid antidepressant action of the *N*-methyl-D-aspartate (NMDA) receptor antagonist ketamine (Li et al., 2010 2011). Therefore, we examined whether mTORC1 signaling facilitated the antidepressant effects of 7,8-DHF and ANA-12 in the treatment of LPS-induced depression-like behavior. Rapamycin (0.2 nmol/L in 2 µL, i.c.v.), a potent mTORC1 inhibitor, was injected immediately before administration of 7,8-DHF (10mg/kg, i.p.) or ANA-12 (0.5mg/kg, i.p.; Figure 3C). One-way ANOVA showed statistical significance for the TST [F(5, 56) = 10.033, *p* < 0.001] and FST [F(5, 55) = 6.525, *p* < 0.001] tests (Figure 3I and J). An infusion of rapamycin significantly blocked the antidepressant effects of 7,8-DHF on LPS-induced depression-like behavior (Figure 3I and J). However, it had no effect on the antidepressant activity of ANA-12 under the same conditions (Figure 3I and J). Rapamycin alone showed no effects in LPS-treated mice (Figure 3I and J). These results implicate mTORC1 signaling in the antidepressant mechanisms of 7,8-DHF, but not those of ANA-12.

### Effects of LPS, 7,8-DHF, ANA-12 on TrkB Phosphorylation in Brain Regions

**Figure 4.**
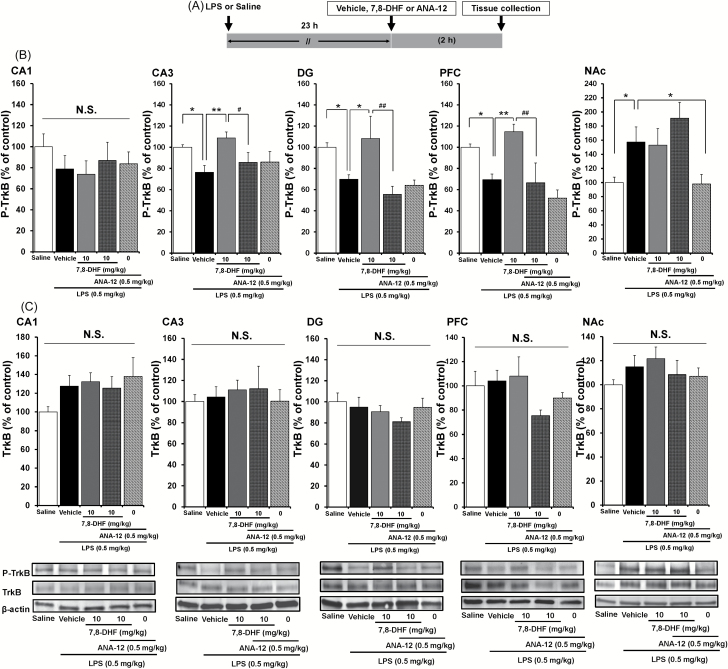
Effects of 7,8-DHF and ANA-12 on LPS-induced changes in phosphorylation of TrkB in the mouse brain. (A): The schedule of treatment. (B): Western blot of p-TrkB protein in the mouse brain. (C): Western blot of total TrkB protein in the mouse brain. The value was expressed as a percentage of that of saline-treated mice. Values represent the mean ± standard error of the mean (n = 5–6). *p < 0.05, **p < 0.01, as compared with the LPS + vehicle group. ^#^ *p* < 0.05, ^##^ *p* < 0.01 as compared with the LPS + 7,8-DHF group. N.S.: not significant. 7,8-DHF: 7,8-dihydroxyflavone; LPS: lipopolysaccharide; TrkB: tropomyosin-receptor-kinase B; p-TrkB: phospho-TrkB.

To clarify whether TrkB activation or inhibition underpins depression-like behavior in LPS-treated mice, we performed immunoblot analyses of TrkB and phosphorylated TrkB (p-TrkB), an activated form of TrkB, in samples from the hippocampus (CA1, CA3, DG), PFC, and NAc. Either 7,8-DHF (10mg/kg, i.p.), ANA-12 (0.5mg/kg, i.p.), or vehicle (10mL/kg, i.p.) was injected 23 hours after LPS or saline administration (Figure 4A). One-way ANOVA showed statistical significance in all of the examined regions [CA1: F(4, 26) = 0.689, *p* = 0.608; CA3: F(4, 25) = 3.297, *p* = 0.030; DG: F(4, 25) = 5.145, *p* = 0.005; PFC: F(4, 25) = 7.248, *p* = 0.001; NAc: F(4, 26) = 4.369, *p* = 0.009]. A single dose of LPS (0.5mg/kg, i.p.) significantly decreased the density of p-TrkB protein in the CA3 (*p* = 0.024), DG (p = 0.044), and PFC (*p* = 0.040), but not the CA1 (*p* = 0.275; Figure 4B). Subsequent treatment with 7,8-DHF (10mg/kg, i.p.) significantly attenuated this LPS-induced reduction of p-TrkB protein in the CA3 (*p* = 0.004), DG (*p* = 0.016), and PFC (*p* = 0.004), but not in the NAc (*p* = 0.318; Figure 4B). Furthermore, ANA-12 significantly blocked the effects of 7,8-DHF on LPS-induced reduction of p-TrkB protein in the CA3 (*p* = 0.034), DG (*p* = 0.002), and PFC (*p* = 0.002; Figure 4B), suggesting a role of TrkB signaling in the mechanisms of 7,8-DHF.

In contrast, a single dose of LPS (0.5mg/kg, i.p.) significantly (*p* = 0.039) increased the density of p-TrkB protein in the NAc (Figure 4B). Subsequent treatment with ANA-12 (0.5mg/kg, i.p.) significantly (*p* = 0.041) attenuated this LPS-induced increase of p-TrkB protein in the NAc (Figure 4B). However, there were no significant changes of TrkB protein in any of the groups (one-way ANOVA; CA1: F(4, 26) = 1.542, *p* = 0.225; CA3: F(4, 28) = 0.839, *p* = 0.514; DG: F(4, 24) = 0.863, *p* = 0.503; PFC: F(4. 26) = 1.359, *p* = 0.280; NAc: F(4, 25) = 0.716, *p* = 0.590; Figure 4C).

### Opposing Effects of 7,8-DHF and ANA-12 on Changes in Dendritic Spines

**Figure 5.**
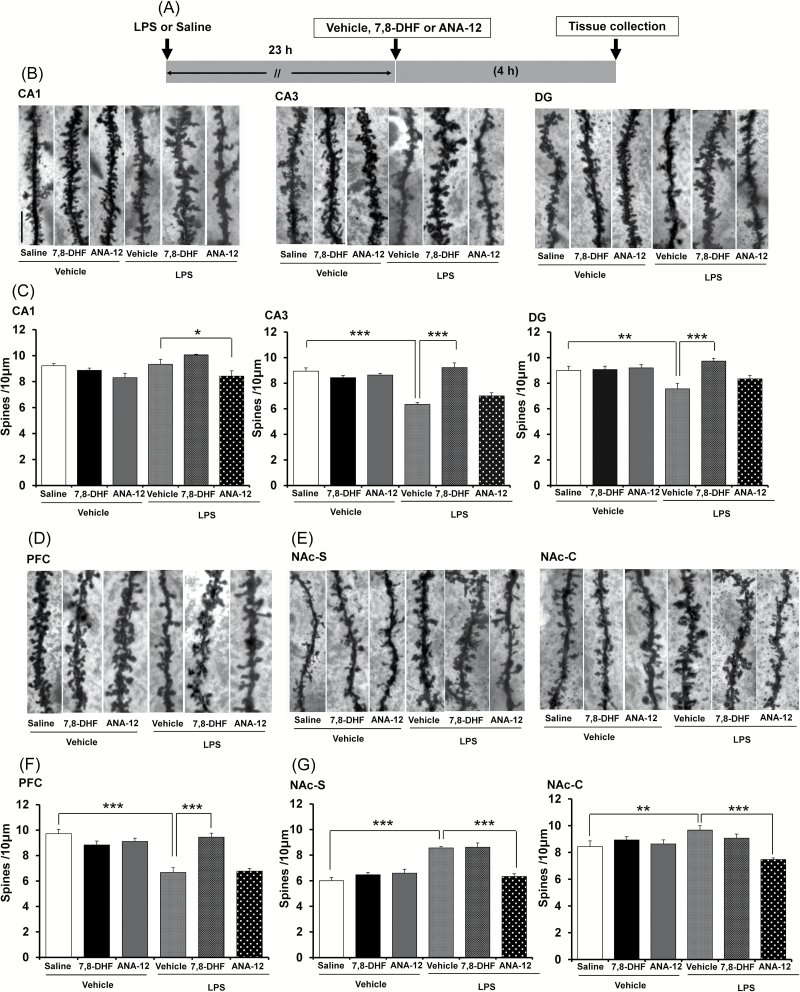
Effects of 7,8-DHF and ANA-12 on LPS-induced changes in spine density within mouse brain. (A): The schedule of treatment. (B): Representative photomicrographs of Golgi-Cox stained pyramidal neurons in the CA1, CA3, and DG of the hippocampus from animals of each group. Scale bar = 10 µm. (C): Values represent the mean ± standard error of the mean (SEM; n = 6). (D): Representative photomicrographs of Golgi-Cox stained pyramidal neurons in the PFC from animals of each group. Scale bar = 10 µm. (F): Values represent the mean ± SEM (n = 6). (E): Representative photomicrographs of Golgi-Cox stained pyramidal neurons in the NAc-shell and NAc-core from animals of each group. Scale bar = 10 µm. (G): Values represent the mean ± SEM (n = 6). *p < 0.05,**p < 0.01, ***p < 0.001 as compared with the LPS + vehicle group. 7,8-DHF: 7,8-dihydroxyflavone; LPS: lipopolysaccharide; DG: dentate gyrus; PFC: prefrontal cortex; NAc: nucleus accumbens.

Changes in dendritic length and spine density in the hippocampus and PFC are thought to contribute to the neurobiology of MDD, and antidepressant treatment is mediated, in part, by blocking or reversing these changes (McEwen, 2007; Duman and Aghajanian, 2012). We examined whether 7,8-DHF or ANA-12 treatment affected changes in the dendritic spines of the hippocampus, PFC, and NAc (Figure 5A). 7,8-DHF (10mg/kg, i.p.), ANA-12 (0.5mg/kg, i.p.), or vehicle (10mL/kg, i.p.) were injected 23 hours after LPS administration (Figure 5A). One-way ANOVA showed statistical significance for all of the examined regions [CA1: F(5, 35) = 5.055, *p* = 0.002; CA3: F(5, 35) = 24.385, *p* < 0.001; DG: F(4, 35) = 5.738, *p* = 0.001; PFC: F(5. 35) = 20.018, *p* < 0.001; NAc-shell: F(5, 35) = 22.410, *p* < 0.001; NAc-core: F(5, 35) = 5.842, *p* = 0.001). A single dose of LPS (0.5mg/kg, i.p.) significantly decreased dendritic spine density in the CA3 (*p* < 0.001) and DG (*p* = 0.003), but not in the CA1 (*p* = 0.788) or within the hippocampus and PFC (*p* < 0.001) of mouse brains (Figure 5B–D and F). Treatment with 7,8-DHF (10mg/kg, i.p.) significantly attenuated this LPS-induced reduction of spine density within the CA3 (*p* < 0.001), DG (*p* < 0.001), and PFC (*p* < 0.001; Figure 5B–D and F). In contrast, treatment with ANA-12 (0.5mg/kg, i.p.) significantly (*p* = 0.033) decreased spine density in the CA1 of LPS-treated mice, indicating that ANA-12 potentiated the effects of LPS in this region (Figure 5B and C). A single dose of LPS (0.5mg/kg, i.p.) significantly increased dendritic spine density in the two areas (shell *p* < 0.001 and core *p* = 0.008) of the NAc (Figure 5E and G). Interestingly, ANA-12 (0.5mg/kg, i.p.) significantly attenuated the LPS-induced increases of spine density in the NAc (shell: *p* < 0.001, core: *p* < 0.001; Figure 5E and G), although it had no effect on reduced spine density in the CA3 (*p* = 0.053), DG (*p* = 0.186) or PFC (*p* = 0.797; Figure 5B–D and F).

## Discussion

This study highlighted both TrkB agonists and antagonists as potential therapeutic drugs for inflammation-induced depression. Our study also revealed a number of major findings: we identified a marked reduction of BDNF protein in the DG, CA3, and PFC, but not CA1, after a single dose of LPS, consistent with a previous study (Guan and Fang, 2006). An infusion of BDNF in the DG and CA3, but not the CA1, promoted antidepressant effects in the rat learned helplessness model (Shirayama et al., 2002), and antidepressant treatment attenuated the decrease of BDNF protein in the PFC after LPS administration (Guo et al., 2014), indicating roles for the DG, CA3, and PFC, but not the CA1, in the antidepressant action of BDNF. This is consistent with alterations seen in BDNF protein in the DG, CA3, and PFC, but not the CA1. In contrast, we found that LPS induced a marked increase in BDNF protein within the NAc, and that bilateral injections of ANA-12 into the NAc promoted antidepressant effects in LPS-treated mice. This finding is similar with a recent report showing that direct infusion of ANA-12 into the NAc completely blocked the ability of phasic stimulation to induce social avoidance (Walsh et al., 2014). The VTA-NAc pathway plays a critical integrative role in reward- and emotion-related behavior (Nestler and Carlezon, 2006). Thus, LPS-induced inflammation produced an opposing effect on BDNF protein levels in the hippocampal, PFC, and VTA-NAc pathways. To the best of our knowledge, this is the first study showing that LPS-induced inflammation increased levels of BDNF in the NAc. Previously, it was reported that intra-VTA BDNF brought about depression-like behavior, while a blockade of BDNF activity in the NAc produced antidepressant-like effects (Nestler and Carlezon, 2006). It is therefore likely that LPS-induced inflammation decreased BDNF in the hippocampus and PFC but increased BDNF in the NAc, resulting in depression-like behavior in mice.

Studies using post-mortem brain samples showed a reduction in BDNF protein in the hippocampus and PFC of psychiatric disorder patients who had committed suicide, compared with non-psychiatric controls (Dwivedi et al., 2003; Karege et al., 2005). A subsequent study showed significantly higher levels of BDNF protein (a 40% increase) in the NAc of patients with depression, relative to controls (Krishnan et al., 2007). These findings in post-mortem brain samples from MDD patients are consistent with our findings in the inflammation-induced mouse model of depression. Thus, it seems that abnormal BDNF levels in the hippocampal, PFC, and VTA-NAc pathways play a causative role in the pathophysiology of MDD, consistent with a recent review (Sun et al., 2013).

Peripheral administration of LPS induces sickness behavior that peaks after two to six hours, then gradually wanes (Dantzer et al., 2008). This behavior results from the activation of pro-inflammatory cytokine signaling in the brain, in response to peripheral LPS injection, and the ensuing depression-like behavior peaks 24 hours post-LPS injection (Dantzer et al., 2008). In this study, we found that 7,8-DHF showed antidepressant effects on LPS-induced depressive behavior, and that ANA-12 could block these effects, indicating a role for TrkB in the action of 7,8-DHF. The antidepressant effect of 7,8-DHF is consistent with previous reports showing that 7,8-DHF could promote both neurogenesis in the hippocampus and marked antidepressant effects (Liu et al., 2010 2012). Surprisingly, ANA-12 alone also conferred antidepressant effects on LPS-induced depression-like behavior, and it is also reported that ANA-12 decreased immobility time in the TST and FST for normal mice (Cazorla et al., 2011). Importantly, it needs to be noted that our findings are therapeutic, but not prophylactic, as 7,8-DHF and ANA-12 were administered into mice previously exposed to LPS. In this model, single doses of conventional antidepressants (SSRI and SNRI) produced no antidepressant effect (Supplementary Figure 2), although pretreatment with conventional antidepressants (SSRIs and SNRIs) conferred a prophylactic effect on LPS-treated mice (Ohgi et al., 2013). It is also reported that a single dose of SSRIs, such as fluoxetine and citalopram, could rapidly activate TrkB phosphorylation in the brain (Rantamäki et al., 2007). Therefore, it is likely that TrkB ligands and classical antidepressants may have differential neurobiological and behavioral effects in depression, although further detailed studies are needed.

Interestingly, rapamycin can block the antidepressant effects of 7,8-DHF, but not those of ANA-12. It has also been reported that the antidepressant-like effect of electroconvulsive therapy in animal models is dependent on reducing BDNF in the VTA, but not on raising hippocampal BDNF expression (Taliaz et al., 2013). Very recently, we found that direct infusion of 7,8-DHF (but not ANA-12) into the hippocampus (CA3 and DG) and PFC and of ANA-12 (but not 7,8-DHF) into the NAc promoted antidepressant effects in the rat learned helplessness model (Shirayama et al., submission), implying that stimulation at TrkB in the CA3 and DG of the hippocampus and in the PFC, as well as blockade of TrkB in the NAc, can confer antidepressant effects. Thus, it is likely that decreased levels of BDNF in the DG and CA3 of the hippocampus and in the PFC, as well as increased levels of BDNF in the NAc, may promote depression-like behavior in rodents. Furthermore, TrkB and subsequent mTORC1 activation are essential to the antidepressant effects of TrkB agonists, whereas mTORC1 signaling may be irrelevant to the antidepressant actions of TrkB antagonists. Moreover, it seems that the blockade of TrkB in the NAc by ANA-12 may be required for its antidepressant effect. A recent study showed that ANA-12 completely blocked the ability of phasic stimulation to induce social avoidance without affecting BDNF amounts in the NAc (Walsh et al., 2014).

Tracking dendritic morphology, we detected opposing changes in spine densities between the the hippocampus, PFC, and NAc. The reduced spine density in the CA3, DG, and PFC is similar to the findings seen in rodents with unpredictable chronic mild stress (Li et al., 2010 2011). Furthermore, BDNF heterozygous (+/-) mice (approximately 50% lower BDNF levels) show a less branched dendritic tree in the CA3 (Magariños et al., 2011) and over-expression of BDNF prevents stress-induced reductions of dendritic branching in the CA3 (Govindarajan et al., 2006), indicating a key role for BDNF in CA3 dendritic spines. Surprisingly, we found increased spine density in the NAc. Additionally, we found that 7,8-DHF attenuated the LPS-induced reduction in spine density in the CA3, DG, and PFC, whereas ANA-12 attenuated LPS-induced increases of spine density in the NAc. A two-photon imaging study showed that BDNF and protein synthesis are crucial to the structural plasticity of single dendritic spines (Tanaka et al., 2008), thus linking changes in BDNF levels in the CA3, DG, PFC, and NAc with altered spine density in these regions. Given that synaptogenesis is a key function in the mechanism of antidepressants (McEwen, 2007; Duman and Aghajanian, 2012), the therapeutic effects of 7,8-DHF and ANA-12 on depression-like behavior, as well as alterations in spine morphology after LPS administration, are of great interest.

Accumulating evidence shows that the HPA axis is hyperactive in patients with MDD, and that the DEX suppression test can effectively quantify dysregulation of this axis (Kathol et al., 1989; Pariante and Miller, 2001). We found HPA system hyperactivity in LPS-treated mice, suggesting that LPS-induced inflammation may alter the neuroendocrine stress axis function. It is therefore likely that LPS exposure may provide an animal model for depression in rodents.

Growing evidence reveals a crucial role for the VTA-NAc reward circuits in the neurobiology of depression (Eisch et al., 2003; Berton et al., 2006; Nestler and Carlezon, 2006; Krishnan et al., 2007; Sun et al., 2013). The VTA-NAc pathway receives strong glutamatergic inputs from several frontal cortical regions, hippocampus, and amygdala (Hyman and Malenka, 2001; Nestler and Carlezon, 2006; Shirayama and Chaki, 2006; Sanacora et al., 2008). Interestingly, it was reported that the NMDA receptor antagonist, ketamine, showed a rapid antidepressant effect via increased BDNF levels (Autry et al., 2011), suggesting a role for BDNF-TrkB signaling in ketamine’s rapid antidepressant response. It would therefore be of great interest to compare the antidepressant effect of TrkB ligands (7,8-DHF and ANA-12) and ketamine in animal models of depression, such as the social defeat stress model and chronic mild stress model. Considering the emerging role of glutamate in the pathophysiology of MDD (Sanacora et al., 2008; Hashimoto, 2009 2013a, 2013b; Zarate et al., 2010; Krystal et al., 2013), it seems that the glutamatergic systems are integral to the emergence of depression-like behavior after inflammation. A recent optogenetic study demonstrated a functional role for the VTA-NAc dopaminergic pathway in encoding reward-related information in depression (Chaudhury et al., 2013). Therefore, it is also likely that both glutamatergic and dopaminergic systems are critical to the development of depression-like behavior after inflammation, although further detailed studies are needed.

Antidepressants, including SSRIs and SNRIs, have been widely used as therapeutic drugs for MDD, although up to two-thirds of patients fail to respond to initial treatment. Chronic treatment with these antidepressants increases BDNF levels in the hippocampus and PFC (Hashimoto et al., 2004; Duman and Monteggia, 2006; Martinowich et al., 2007; Hashimoto, 2010; Duman and Aghajanian, 2012), confirming BDNF-TrkB signaling as part of the therapeutic mechanism of antidepressants. Finally, we would like to propose the use of TrkB ligands as potential therapeutic drugs for MDD. Post-mortem data from depressed patients showed that depression is associated with a decrease in BDNF levels in the hippocampus and PFC, and an increase of BDNF in the NAc (Dwivedi et al., 2003; Karege et al., 2005; Krishnan et al., 2007; Sun et al., 2013). From our results, TrkB agonists could represent effective therapeutic drugs for MDD patients with decreased BDNF levels in the hippocampus and PFC. It has been shown that BDNF in the VTA, but not the hippocampus and PFC, helps to mediate the antidepressant-like effect of electroconvulsive therapy, which is widely used in the treatment of refractory MDD (Kathol et al., 1989). This finding adds weight to the theory that TrkB antagonists can act as therapeutic agents for treatment-resistant MDD patients who show increased BDNF-TrkB signaling in VTA-NAc pathways. Finally, the use of novel biomarkers (e.g., blood levels of proBDNF or BDNF) for MDD could prove invaluable in detecting the antidepressant efficacy of TrkB agonists/antagonists (Hashimoto, 2010, 2014a, 2014b). Further detailed studies will be needed to confirm this hypothesis.

In conclusion, our study shows that LPS-induced inflammation caused depression-like behavior, as well as alterations in BDNF protein and spine density within the hippocampus, PFC, and NAc. Furthermore, antidepressant effects were shown on LPS-induced depressive behavior by normalizing altered dendritic spines in the hippocampus and PFC with TrkB agonist 7,8-DHF and in the NAc with antagonist ANA-12. Therefore, abnormal BDNF-TrkB signaling in the hippocampus, PFC, and NAc may play a role in inflammation-induced depression. Finally, in MDD, TrkB agonists and TrkB antagonists could act as potential therapeutic drugs for patients with lower BDNF levels in the hippocampus and PFC, and those with higher levels of BDNF in the NAc.

## Supplementary Material

For supplementary material accompanying this paper, visit http://www.ijnp.oxfordjournals.org/

## Statement of Interest

Dr Hashimoto has served as a scientific consultant to Astellas and Taisho, and he has also received research support from Abbvie, Dainippon Sumitomo, Otsuka, and Taisho. The other authors report no biomedical financial interests or potential conflicts of interest. Dr Shirayama has received research support from Eli Lilly, Eisai, MSD, Otsuka, Pfizer, Taisho, Takeda, and Mitsubishi-Tanabe.
